# Home-Leaving Patterns Among Middle Eastern-Origin Youth in Sweden: The Influence of Origin, Generation, and Neighbourhood

**DOI:** 10.1007/s10680-026-09776-8

**Published:** 2026-06-19

**Authors:** Rami Zalfou, Anna Tegunimataka

**Affiliations:** Department of Economic History, Lund University, Lund, Sweden

**Keywords:** Home leaving, Middle Eastern immigrants, Second-generation immigrants, Competing risks analysis, Sweden

## Abstract

This study examines home-leaving patterns among Middle Eastern (ME) immigrants and youth with ME backgrounds in Sweden using population register data for individuals aged 17 to 35 between 1998 and 2022. Applying competing risks models, we analyse transitions from the parental home to independent living while accounting for marriage as an alternative pathway. The results show that youth of ME origin are less likely than native Swedes to leave the parental home for independent living and more likely to transition directly into marriage. These differences are particularly pronounced among the second generation and among women. Individual and parental socio-economic characteristics explain only a small share of the observed gaps between immigrants and natives, while neighbourhood unemployment is strongly associated with lower transition rates to independent living. Overall, the findings highlight persistent differences in the timing and pathways of leaving the parental home between immigrant-origin youth and the native population.

## Introduction

Leaving the parental home is a key milestone in the transition to adulthood, often associated with growing economic independence and social autonomy. In Western European societies, this transition tends to occur earlier and more frequently than in non-Western contexts. However, immigrant youth often follow distinct home-leaving trajectories influenced by a combination of economic, cultural, and social factors (Gillespie et al., 2020; Kleinepier & de Valk, 2017; Zorlu & Mulder, 2011). These differences are central to a broader understanding of migrant incorporation, which extends beyond the labour market into areas such as housing, family formation, and social inclusion. In this sense, leaving the parental home is important not as a direct measure of cultural integration, but as an indicator of residential independence and unequal transitions to adulthood.

In Sweden, where establishing independent living is the predominant route out of the parental home, remaining with parents into one’s mid-thirties indicates a marked postponement of this transition and thus a significant divergence in life-course trajectories. In this paper, the transition to independent living is defined as the move from the parental home into a separate household, reflecting patterns typical of the Swedish context, while explicitly excluding family formation events such as marriage. This conceptualisation aligns with Sweden’s institutional and cultural setting, where leaving home tends to occur relatively early and is often facilitated by welfare support and accessible housing. The analysis focuses on youths of Middle Eastern (ME) origin within this framework, a population shaped by cultural and economic backgrounds in which norms surrounding departure from the parental home differ substantially.

Swedish youth, on average, leave the parental home earlier than youths in other EU countries (Eurostat, 2024). However, these patterns do not necessarily extend to all segments of the population. Sweden has seen a significant increase in immigration from the ME region in recent decades, and youths from these backgrounds likely have distinct home leaving patterns. Such differences may reflect persistent cultural expectations around family and co-residence, as well as unequal access to economic resources, often linked to higher unemployment and lower income among the parental generation (OECD, 2023). These factors may also be different across migrant generations, with first-generation youth often facing more immediate constraints and second-generation often find themselves balancing their parents’ traditions with the expectations of the host country, but also by gender. Girls from ME backgrounds may face stronger expectations around family responsibility, protection, or modesty (Berry et al., 2006), which can delay or complicate independent living. Boys, on the other hand, may experience greater freedom but also increased pressure to contribute economically to the household (Berry et al., 2006). These patterns can also differ depending on country of origin, reflecting diverse cultural norms, migration histories, and integration.

An important consequence of the increasing migration to Sweden has been the emergence of large, ethnically concentrated neighbourhoods in major cities. Youth from ME backgrounds often grow up in these areas, where strong ties to the co-ethnic community potentially has an influence on their home leaving decisions. The presence of a large, cohesive immigrant community may ease the pressure to leave home by providing strong social support networks and reinforcing norms around intergenerational co-residence (Portes & Rumbaut, 2001; McAvay & Pailhé, 2022). In contrast, youth living in areas with smaller or more dispersed co-ethnic communities may face greater cultural dissonance and instead pressure to adapt to mainstream expectations of autonomy (McAvay & Pailhé, 2022).

Using Swedish population register data from 1998 to 2022; this paper examines the home-leaving patterns of individuals aged 17 to 35. We track transitions from living with parents to either independent living or to living in a married household, focusing on first- and second-generation individuals from ME backgrounds. We compare these patterns to those of native-born Swedes with two Swedish-born parents, as well as to selected European immigrant groups. Our analysis employs competing risks models to estimate the timing and likelihood of leaving the parental home, accounting for marriage as a competing destination. We include measures of generational status, gender, post-secondary education and employment status, own and parental socio-economic status, and contextual factors like the shera of migrants vs. natives in the local area as well as unemployment levels in the local area.

Our results show that youth from ME backgrounds are less likely to leave the parental home to live independently and are more likely to transition directly from the parental home to living in a married household. This pattern is particularly apparent among second generation immigrants with two foreign-born, even when compared with ME-born youth that arrive in Sweden as children. This is a notable finding, as it shows that lower rates of independent home-leaving do not decline monotonically across immigrant generations. We also find this pattern to be stronger among women as compared to men.

Overall, we find that individual and parental socio-economic conditions are not major drivers of these gaps, and that they persist when controlling for individual contextual factors and socio-economic conditions, as well as parental socio-economic conditions, and when looking across parental income quantiles. Our findings point to persistent intergenerational differences in pathways out of the parental home that are not readily explained by the observed socio-economic and neighbourhood factors included in our models. This highlights home-leaving as an important domain for understanding broader processes of immigrant incorporation and adaptation across generations.

When looking at the role of the neighbourhood, we find that both the native share of the population in the local neighbourhood, and the share unemployed in the local neighbourhood, matter for the gaps in transitions between immigrants and natives. These findings suggest that norms around independence and early marriage persist across generations and are reinforced by local community contexts. However, there is also evidence of generational divergence, with youth who arrived in Sweden at younger ages showing more alignment with majority Swedish patterns of home-leaving as compared to later arrivals.

This study contributes to the literature in three ways. First, it provides new evidence on home-leaving patterns among immigrant-origin youth in Sweden using population-wide longitudinal register data covering more than two decades. This allows us to document differences in the timing and pathways out of the parental home between ME-origin youth and native Swedes with a high degree of precision. Second, the analysis distinguishes between several immigrant generations, including detailed age-at-arrival categories among the foreign-born and a distinction between second-generation youth with one or two foreign-born parents. This approach allows us to examine whether patterns of home-leaving converge or diverge across immigrant generations. Third, the study examines how neighbourhood context relates to these transitions by incorporating postcode-level measures of immigrant concentration and unemployment. By linking individual life-course transitions to neighbourhood characteristics, the paper provides additional insight into how local environments are associated with differences in residential independence among immigrant-origin youth.

## Literature Review

Research on home-leaving explores a number of factors that influence when young adults move out from their parental home. This transition is driven by a combination of personal decisions, economic conditions, and cultural expectations (Marini, 1984). Important life events, such as securing employment, entry into higher education, and marriage, all can give the opportunity and motivation to move out among young people (Goldscheider & Goldscheider, 1987). Structural conditions, such as housing prices and governmental support for youth can either delay or speed up the process (Marini, 1984). However, moving out triggers can vary significantly depending on the individual’s background, access to resources, and expectations from the family. Cultural norms are particularly influential for youth from immigrant backgrounds, where ideas about family responsibilities, independence, and gender roles may contrast with those of natives in the host country (de Valk & Liefbroer, 2007).

### Structural and Economic Conditions

In the Swedish context, the transition out of the parental home is supported by the social democratic welfare regime, with a stable age at leaving the parental home even during economic downturns and spikes in youth unemployment rates (Buchmann & Kriesi, 2011; Dribe & Stanfors, 2005). Overall, the age at leaving the parental home changed little in Sweden over the second half of the 20th century, despite fundamental social and economic changes (Dribe & Stanfors, 2005). However, recent literature shows that economic setbacks, such as becoming unemployed, are associated with increased return to the parental home (Olofsson et al., 2020). This underscores the importance of job availability and wages, as financial security increases likelihood of living independently (Olofsson et al., 2020). Parental resources also matter for young people’s home-leaving opportunity as many young need the financial support of their parents to cover housing and living costs (Zorlu & Mulder, 2011). Economic conditions are especially relevant for migrant youth. Hoolachan et al. (2017) highlight how young people face distinct financial challenges, such as higher unemployment and less job stability, which could hinder their ability to afford independent housing. This is frequently the case for immigrant youth and youth with immigrant background, who tend to experience greater economic uncertainty, higher unemployment, and labour market discrimination in many contexts (Gorodzeisky & Semyonov, 2017; Carlsson & Rooth, 2007). The geographic concentration of immigrants in large metropolitan areas such as Stockholm, Gothenburg, and Malmö, may also influence home-leaving patterns. Such urban housing markets are characterized by limited availability of rental housing and relatively high housing prices compared to smaller cities and towns.

In response to these challenges, education often becomes a central focus. Young people from immigrant backgrounds often prioritize higher education (Salikutluk, 2016; Jonsson & Mood, 2023), which can be an important path toward economic mobility in the host country (Borjas, 2006). In Sweden, where student allowances and housing options are available, attending university may provide an opportunity to leave the parental home.

### Cultural Influences, Migrant Contexts and Gender

Cultural factors play an important role in influencing the home-leaving behaviour of migrant youth, particularly for those with origins in the Middle East and NorthAfrica (ME). In much of the ME region, leaving the parental home is closely tied to marriage (Sonneveld, 2019). For women, marriage is often a prerequisite due to cultural, religious, and legal restrictions (Gebel & Heyne, 2014, p. 228), while men are typically expected to be able to provide financially for a new household before marrying (Kovacheva et al., 2018). Over recent decades, rising participation in higher education and delayed marriage have pushed the average age of home-leaving higher in the ME region (Carmichael, 2011). Nonetheless, large gender gaps remain, with women marrying and thus leaving home, several years earlier than men (Koç, 2007).

These cultural norms can carry over to migrant populations, influencing behaviour in the host country. Compared to their native peers, migrant youth often face different circumstances and expectations. Immigrant youth may feel obligated to remain in the parental home to contribute financially or logistically to the household (van Hook & Glick, 2007). They may also assume unique family responsibilities, such as language brokering, helping parents navigate linguistic and bureaucratic barriers (Villanueva & Buriel, 2010; Melander & Shmulyar Gréen, 2023). These roles can delay departure from the parental home as their presence becomes essential to family functioning, while intergenerational transmission of values further influence home-leaving patterns. De Valk and Liefbroer (2007), studying migrant families in the Netherlands, find that in many communities, especially those from collectivist cultural backgrounds, remaining at home until marriage or another major life milestone is a deeply embedded norm. This is not solely due to economic constraints but also reflects a cultural emphasis on familial support and unity. Gender norms play a role as well: young women in migrant families often face stronger expectations to stay at home, reflecting traditional caregiving roles even in context that could facilitate greater independence (Yalim et al., 2023).

Patterns in previous research are not uniform. Using Dutch administrative data, Zorlu and Mulder (2011) report that Turkish and Moroccan youth leave home at younger ages than native Dutch, most often for union formation and also for independent living, even though later home-leaving is common in the countries of origin. The authors point to factors such as the availability of welfare supports and migrant youths’ position between origin and host-country norms as possible explanations.

Migrants’ generational status also matters. Rumbaut (2004) distinguishes the second generation with two foreign-born parents, the 2.5 generation with one foreign-born and one native-born parent, the third generation with two native-born parents, and decimal generations among the foreign-born (for example, 1.75 for arrivals at ages 0–5 and 1.5 for arrivals at ages 6–12). Children with a native-born parent tend to have greater fluency in the official language and stronger identification with the host society, which can facilitate earlier home-leaving than among first-generation youth. Recent United States evidence links these gradations to home-leaving: Gillespie et al. (2020) find the highest hazards of departure among third-plus generation youth, followed by the second generation, then the 1.75 and 1.5 generations, with parental region of origin and non-English home language further reducing exit risks, particularly among youth with Latin American-born parents.

In Canada, analyses show that both the timing and destination of exits differ by immigrant generation and visible-minority status, although not in a uniform or strictly monotonic way across groups (Haan et al., 2023). Yet it remains unclear whether such nuanced generational categories correspond to consistent differences in when young people leave the parental home in European settings.

In this study, we distinguish between two main routes out of the parental home: on the one hand, independent living (including nonmarital cohabitation), and on the other, direct transition into marriage. The distinction is crucial because the prevalence and social significance of cohabitation and marriage differ across societies and migrant generations. In Sweden and other Northern European countries, leaving the parental home typically occurs through independent living rather than through marriage. Cohabitation and solo living constitute the dominant pathways out of the parental home among native youth, while direct transitions into marriage are comparatively rare (Billari & Liefbroer, 2007; Hiekel et al., 2014). This pattern is also visible in our data: among native Swedes in our sample, only around 1.5% leave the parental home directly into marriage (Table 1). This institutional context motivates our distinction between independent living and direct transitions into marriage. Immigrants and their descendants follow distinct union pathways: in the United Kingdom, origin groups differ in propensities to cohabit or marry (Hannemann & Kulu, 2015) and in the Netherlands, union choices among the Turkish and Moroccan second generation are closely tied to social embeddedness and networks (Huschek et al., 2011).

**Table 1:** Descriptive statistics

Origin		Middle east						Eastern Europe					
Generation	Natives	2.5	2	1.75	1.5	1.25	1	2.5	2	1.75	1.5	1.25	1
Observations	5,651,598	16,125	114,966	39,553	45,687	16,332	3,231	32,003	27,584	9,443	7,879	3,965	525
Individuals	886,374	2,244	14,986	6,276	8,415	3,738	684	4,655	3,676	1,377	1,203	594	91
Sex (%)													
Male	52	52	52	54	56	60	55	52	52	53	53	56	43
Female	48	48	48	46	44	40	45	48	48	47	47	44	57
Status at the end of observation (%)													
Living independently	97.8	92.5	77.8	87.7	85.3	81.5	88.4	96.4	91.4	93.0	91.3	83.6	95.5
Married	1.5	3.1	13.8	8.3	10.6	13.1	8.7	0.9	2.2	3.8	6.1	5.0	3.4
In parental home	0.7	4.4	8.4	4.0	4.1	5.4	2.9	2.7	6.4	3.2	2.6	11.4	1.1
Post-secondary education (%)	9.5	15.8	23.3	19.2	16.8	14.5	13.8	17.9	23.4	19.4	25.2	18.2	17.1
In employment (%)	42.0	37.2	47.3	39.2	40.1	38.8	26.6	36.3	34.7	34.2	33.2	37.1	23.0
Total earned income (mean, SEK)	73,100	73,200	117,000	87,800	89,500	96,500	61,800	68,000	72,400	70,400	67,600	88,000	45,400
Occupation (%)													
Farming, industrial, and transport workers	7.6	2.5	2.7	3.0	3.2	2.6	1.5	5.1	3.2	4.8	4.9	3.3	1.5
Managers and officials	0.06	0.17	0.22	0.19	0.16	0.11	0.00	0.08	0.02	0.12	0.14	0.00	0.00
Military personnel	0.13	0.13	0.04	0.03	0.02	0.03	0.00	0.13	0.08	0.15	0.01	0.03	0.00
Professionals	2.6	2.9	3.2	3.5	2.8	1.3	3.2	3.5	3.5	4.0	4.5	1.8	2.3
Service workers	20.6	19.3	17.6	20.3	18.4	12.0	13.6	18.8	17.0	17.4	17.1	10.0	15.4
Unknown	69.0	75.1	76.2	72.9	75.4	83.9	81.6	72.3	76.3	73.5	73.3	84.8	80.1
Income quartile (%)													
Quartile 1	21.5	30.4	27.1	31.0	31.7	35.9	42.2	28.7	33.4	33.3	34.3	38.3	41.8
Quartile 2	23.7	20.0	14.4	18.3	17.5	15.7	19.9	21.9	19.2	21.3	20.6	12.0	23.8
Quartile 3	27.2	23.6	20.4	21.7	21.4	17.1	20.1	25.3	23.0	21.6	22.8	20.2	20.0
Quartile 4	27.5	25.9	38.0	29.0	29.4	31.3	17.8	24.0	24.4	23.8	22.4	29.5	14.3
Father in employment (%)	90.8	79.0	72.0	68.1	63.6	45.1	56.2	80.0	74.5	77.9	79.7	79.7	77.5
Mother in employment (%)	90.9	86.1	68.3	70.0	63.1	41.2	54.7	85.4	82.0	82.6	80.7	74.6	83.8
Total parental income (mean, SEK)	610,900	546,200	393,300	365,800	313,800	221,400	291,900	579,800	520,300	513,600	515,800	514,200	604,900
Parental income quartile (%)													
Quartile 1	10.5	19.0	38.5	41.7	49.8	67.8	56.7	18.3	22.7	22.9	25.0	28.4	25.1
Quartile 2	24.5	25.7	29.3	30.4	28.6	17.1	24.0	24.6	26.9	30.2	29.6	27.9	22.8
Quartile 3	31.8	25.7	18.5	17.7	14.6	9.3	11.1	25.1	24.2	23.6	21.3	21.1	20.0
Quartile 4	33.1	29.6	13.6	10.2	7.0	5.8	8.2	31.9	26.2	23.2	24.0	22.5	32.0
Number of postcodes	10,439	2,532	4,394	3,415	3,546	2,161	857	4,291	3,207	1,776	1,558	923	171
Postcode categories (%)													
Native > = 75%	93.4	75.7	43.4	48.8	42.1	37.3	48.7	86.7	70.0	65.7	65.7	50.8	66.9
Unemployed < 25%	59.7	63.9	54.2	62.4	62.5	63.3	75.5	65.2	66.5	67.3	68.9	63.8	78.1
Native > = 75% & Unemployed < 25%	37.2	26.0	18.3	17.3	14.9	13.2	12.3	29.4	22.5	20.1	19.7	16.5	12.2
Native > = 75% & Unemployed > = 25%	56.2	49.7	25.0	31.5	27.3	24.1	36.5	57.4	47.5	45.6	46.0	34.3	54.7
Native < 75% & Unemployed < 25%	3.0	10.0	27.4	20.3	22.6	23.5	12.2	5.4	11.0	12.6	11.4	19.7	9.7
Native < 75% & Unemployed > = 25%	3.6	14.3	29.2	30.9	35.2	39.2	39.1	7.9	19.0	21.7	22.9	29.5	23.4

The table shows descriptive statistics for the sample used in the analysis. Measures are evaluated using all observations, except for sex and status at the end of observation which are evaluated based on individuals

### Neighbourhood and Ethnic Community Influence

The neighbourhood ethnic composition may have a direct influence on home-leaving decisions, yet few studies have investigated the direct influence of neighbourhood composition on home-leaving behaviour (McAvay & Pailhé, 2022). The neighbourhood is important for the socialization of youth, as it influences behaviours, peer interactions, exposure to role models, access to social capital and social control. These factors contribute to the development of behaviours, attitudes, and aspirations (Sampson, 2012). Immigrant youth that live in neighbourhoods with a large co-ethnic group is more likely to have their day-to-day interactions within the ethnic groups (van Tubergen & Maas, 2007) and a greater exposure to the norms and expectations of that particular group also regarding the timing of life events such as leaving the parental home Moreover, as highlighted by research on residential behaviour among international migrants (e.g. Bowes et al., 1997; Aradhya et al., 2017), the presence of co-ethnics and family members in the neighbourhood significantly affect residential choices. Migrants often choose to settle near people from their country of origin upon arrival, with family proximity being important in location decisions. These preferences are strengthened by cultural norms of family solidarity, which are often stronger among non-Western migrant groups compared to the native-born population (Dykstra & Fokkema, 2012). Such norms can foster a reluctance to move away from family members, reinforcing intergenerational co-residence and delaying home-leaving among youth.

McAvay and Pailhé (2022) extend this discussion to the French context, showing that neighbourhood composition plays a role for home-leaving decisions among immigrant youth. Their study finds that young people from immigrant backgrounds living in immigrant-dense areas tend to leave home later, net of individual, family, and contextual controls. The association differs by origin and gender: for example, the negative link is especially clear for leaving to unmarried cohabitation, while for some groups (e.g., North African women) higher neighbourhood immigrant shares are linked to more exits into marriage. These findings highlight the importance of considering local residential environments when analysing the transition to adulthood, particularly among immigrant populations.

The neighbourhood and its composition also matter for the opportunities in terms of jobs and education, as well as finding independent housing. There may also be discrimination directed towards those residing in a particular neighbourhood which may impact their possibilities of finding housing elsewhere (Pager & Shepherd, 2008).

Summarizing the literature reviewed above, prior studies point to clear differences in home-leaving patterns between native youth and youth with ME backgrounds. Youth from ME backgrounds, especially women, tend to leave the parental home later due to cultural expectations that link home-leaving to marriage and emphasize family responsibilities. Young women in migrant families often face stronger expectations to stay at home, while men’s departure is more often linked to their ability to provide economically. Patterns across migrant generations are less clear, as previous studies reach different conclusions. Nonetheless, some find that second-generation youth, particularly those with one native-born parent, tend to leave home earlier than first-generation peers, though often still later than natives, with pathways varying by context and origin, although fewer studies have focused on the European context. Neighbourhoods with a high concentration of co-ethnics can reinforce cultural norms and family ties, making youth more likely to remain at home longer. In these areas, limited access to housing and jobs may also delay the possibility of moving out.

The literature leads to four expectations that structure our empirical analysis. First, we expect ME-origin youth to be less likely than native Swedes to leave the parental home for independent living. Second, we expect these gaps to be larger among women than among men. Third, we expect home-leaving patterns to vary across migrant generations, although this variation is not necessarily monotonic. Fourth, we expect neighbourhood context, particularly local labour-market disadvantage and residential concentration, to be associated with slower transitions to independent living. We also examine heterogeneity by country of origin, but treat this part of the analysis as exploratory, since differences between origin groups may reflect a combination of family norms, migration histories, selection, and compositional differences across generations, rather than a single expected ranking across specific origin countries.

## Data and Methods

We use Swedish longitudinal administrative data that provides annual observations on all individuals legally residing in Sweden. The data includes comprehensive demographic characteristics, such as age, sex, country of birth, and place of residence, as well as detailed information on individuals’ linked parents. For individuals and their parents, we observe educational attainment, labour market participation, income, and occupation on a yearly basis, allowing us to account for a wide range of background variables that may influence young people’s transition out of the parental home. Our analytical sample consists of individuals born between 1981 and 1992. These cohorts are tracked from age 17 until the year they turn 35 (from the year 1998 until 2022), providing a long observational window that captures both early and delayed transitions out of the parental home. Because leaving the parental home is typically completed much earlier in Sweden, observing individuals through age 35 allows us to distinguish delayed transitions from more persistent non-departure from the parental home.

The primary outcome of interest is the timing and type of departure from the parental home. We define “leaving the parental home to live independently” as a situation in which the individual no longer resides with either parent and is not registered as part of a married household. This definition captures individuals who move out to live alone, with roommates, or with a partner outside of marriage. Due to limitations in the administrative data, we are unable to observe cohabitation directly; however, since cohabiting unions are common in Sweden and indistinguishable from other independent living arrangements in register data (unless the partners have common children), we classify such cases as independent living.

We focus primarily on the transition from the parental home to independent living, distinguishing it from direct transitions into marriage. The latter is treated as a competing pathway, as individuals may exit the parental home either to live independently or to form a marital household. This distinction is crucial, particularly in the context of intergenerational differences and cultural variation, where norms around marriage and cohabitation may differ significantly (discussed above). Accordingly, our analysis adopts a competing risks framework to model these two mutually exclusive pathways out of the parental home.

Because cohabitation cannot be consistently identified in the register data unless partners have common children, we do not attempt to distinguish cohabiting unions from other forms of independent living in the main analysis. As a result, transitions classified as independent living include solo living, shared housing, and non-marital cohabitation.

We focus on immigrants and their offspring with origin in the ME region and include individuals from the top seven sending countries in that region (Afghanistan, Iraq, Iran, Lebanon, Somalia, Syria, and Turkey). We split both the immigrant and the second-generation samples into several groups based on age at arrival in Sweden for immigrants and based on parental origin for the second generation. The sample is split into six generations:

Generation 1: those who arrive in Sweden at age 17.^1^

Generation 1.25: those who arrive at ages 13 to 16.

Generation 1.5: those who arrive at ages 6 to 12.

Generation 1.75: those who arrive at ages up to 5.

Generation 2: those who are born in Sweden to two foreign-born parents.

Generation 2.5: those born in Sweden to one foreign-born and one Sweden-born parent.

We compare the outcomes in these groups to those of native-born Swedes with two Sweden-born parents, and we also use a comparator group of migrants originating in Eastern European countries (Poland, Russia, Romania, Bulgaria, Hungary, and Ukraine), which constitute important immigrant sending countries in Sweden.

We use competing risks models which measure the relative risk of leaving home into independent living, with transitioning directly to marriage treated as a competing risk. This method follows the Fine and Gray (1999). The model allows for estimating the effects of different covariates on the cumulative probability of the event of interest while accounting for competing risks. The hazard function is defined as:

$$\:{\lambda\:}_{k}\left(t|X\right)=\:\underset{\varDelta\:t\to\:0}{\mathrm{lim}}\frac{\mathrm{Pr}\left(t\le\:T<t+\varDelta\:t,\:\:D=k\:\right|T\ge\:t,\:X)}{\varDelta\:t}$$

Where T is the event time and $\:k$ is the event type. The regression equation takes the following form:

$$\:{\lambda\:}_{k}\left(t|X\right)=\:{\lambda\:}_{k0}\left(t\right)\:\mathrm{e}\mathrm{x}\mathrm{p}({{\upbeta\:}}_{1}{\mathrm{X}}_{1}+\:{{\upbeta\:}}_{2}{\mathrm{X}}_{2}+\dots\:)$$

Where $\:{\lambda\:}_{k}\left(t|X\right)$ is the subdistribution hazard for cause $\:k$ at time $\:t$ given the covariates in $\:X$. $\:{\lambda\:}_{k0}\left(t\right)$ is the baseline subdistribution hazard for cause $\:k$, and the $\:X{\prime\:}s$ refer to the included covariates.

We treat the native group with two native-born parents as our baseline and add a dummy variable for each region of origin and generation. We control for a set of individual factors, which include post-secondary education, employment status, total yearly income, and occupation. We further control for parental socio-economic status by adding controls for the parental income (total of both parents’ incomes), and the employment status and occupation of each parent. Finally, we control for county fixed effects, and municipality fixed effects in an alternative specification, to capture geographic heterogeneity.

To explore the role of the neighbourhood, we construct variables at the postcode level that capture the share of Sweden-born individuals in each postcode, and the share of unemployed individuals in each postcode. We construct two variables at the postcode level to capture the conditions in the local neighbourhood: a dummy variable that takes the value 1 if the share of natives is at 75% or more, and 0 otherwise, and a dummy variable that takes the value 1 if the share of individuals who are unemployed is at 25% or more, and 0 otherwise. While the vast majority of natives Swedes live in neighbourhoods with a native share of 75% or more, less than half of the ME population in our samples live in such neighbourhoods. 2

While Swedish administrative registers provide high-quality longitudinal data, several limitations should be acknowledged. First, cohabitation cannot be directly identified unless partners have a common child, meaning that some partnership transitions are classified as independent living. Second, although residential registers are generally accurate, short-term or temporary moves (particularly among young adults) may occasionally go unrecorded. Third, the registers capture formal residence rather than actual living arrangements, which may differ in some cases. An additional limitation is that, while the register data allow us to control for a broad set of parental socio-economic characteristics, our modelling do not capture several dimensions of parental migration background that may vary systematically across the generational categories we study. These include, for example, parental arrival cohort, migration trajectory, and more detailed parental origin composition. Such factors may contribute to the observed differences in home-leaving behaviour across generations. The estimates should therefore be interpreted as documenting differences in pathways out of the parental home across origin-generation groups, rather than as isolating the causal effect of integration across generations.

The analysis relies on the Fine and Gray (1999) competing risks framework, which models the subdistribution hazard of each event type. A key implication of this approach is that individuals remain in the risk set even after they experience a competing event. For example, individuals who exit the parental home through marriage are retained in the risk set for independent living, albeit with appropriately weighted risk contributions (Fine & Gray, 1999). This feature allows the model to estimate the cumulative incidence of independent living while correctly accounting for marriage as a competing pathway, rather than censoring such cases. At the same time, it means that the risk sets used in the estimation do not correspond exactly to the set of individuals who are “at risk” in a substantive sense after experiencing a competing event. Readers should therefore interpret the coefficients as pertaining to differences in the relative incidence of outcomes, not to literal hazards conditional on remaining unmarried or at home.

Another limitation of the Fine and Gray model is that it is restricted to the first observed transition and does not capture subsequent moves (for instance, returning to the parental home or moving between independent living and cohabitation). Our focus is therefore on the timing and type of first departure from the parental home. The assumption of mutually exclusive and exhaustive event types simplifies the complexity of residential trajectories, while events such as international migration or informal cohabitation are not accounted for in the models.

Overall, the Fine & Gray approach is well suited to our research question, as it allows us to directly compare the relative likelihood of leaving the parental home through independent living versus marriage, while accounting for the fact that one type of transition precludes the other. However, it also entails that results should be interpreted as differences in cumulative incidence across groups, rather than as instantaneous risks in the presence of dynamic, sequential life-course events.

## Results

### Descriptive Statistics

Table 1 shows the descriptive statistics for the analytical sample. The majority of individuals in the sample transition to living independently by the end of the observation period at 35 years of age (97.8% in the native group). Though the proportion differs by region of origin and by generation. We find that the proportion is generally lower in the ME origin sample, measuring between 81% and 88% in the first generation and declining to 78% in generation 2. The mixed 2.5 generation on the other hand appears to be more similar to the natives with a share at 92%. The share transitioning directly from the parental home to living in a married household is at only 1.5% in the native sample and appears to be higher in the ME first generation measuring between 9% and 13% and increasing to 14% in the second generation but remains at a much lower 3% in the 2.5 generation. The proportion remaining in the parental home without any transitions is at below 1% in the native sample, and measures between 3% and 5% in the first generation, increasing to 8% in the second. The ME first-generation transition shares are similar to those in the Eastern European first generation, but they diverge in the second generation, with a comparatively high share of ME-origin second generation youths transitioning directly to marriage.

The share of individual observations with post-secondary education measure at 9.5% in the native sample and appear to be elevated in the first-generation ME-origin groups, measuring between 14% and 19%, and increasing to 23% in the second generation. The second generation also stands out in the high share in employment (47%) as compared to natives (42%), and in their average yearly incomes (117,000) as compared to the corresponding value in the native group (73,100), and as compared to the figure in the second-generation Eastern European sample (72,400).

The patterns in parental income contrast with own earned income, with the native sample having the highest mean of parental income at 610,900, while the first- and second-generation ME samples have lower values ranging from 221,400 to 393,300. These figures also contrast with the corresponding values in the Eastern European sample, where parental income is much closer to the native mean and consistently above 500,000. These patterns are also reflected in the disparities seen in the parental income quartiles between ME migrants and the native sample, and in parental employment figures.

### Differences Across Groups

Table 2 presents the results from competing risks regressions, estimated separately for males and females. The coefficients are reported as subdistribution hazard ratios (SHRs), which have been exponentiated for easier interpretation. A coefficient of 1 indicates no difference in the subdistribution hazard compared to the reference group. A coefficient below 1 indicates a lower subdistribution hazard, meaning the group experiences the event of interest at a slower rate over time compared to the reference. For example, a coefficient of 0.5 suggests that the group’s subdistribution hazard is half that of the reference group, although this does not mean the absolute risk is exactly halved.

**Table 2:** Coefficients of competing risks regression

	Male		Female	
	(1)		(2)	
Reference group	Natives		Natives	
Origin	ME	EEURO	ME	EEURO
Generation				
2.5	0.824^***^	0.846^***^	0.695^***^	0.836^***^
	(−6.81)	(−8.58)	(−12.16)	(−9.28)
2	0.511^***^	0.701^***^	0.376^***^	0.629^***^
	(−52.88)	(−15.62)	(−62.45)	(−18.65)
1.75	0.690^***^	0.770^***^	0.505^***^	0.696^***^
	(−19.79)	(−6.84)	(−28.20)	(−8.54)
1.5	0.657^***^	0.648^***^	0.464^***^	0.634^***^
	(−23.45)	(−10.02)	(−24.81)	(−9.22)
1.25	0.653^***^	0.606^***^	0.458^***^	0.576^***^
	(−15.10)	(−8.85)	(−19.30)	(−9.05)
1	0.670^***^	0.934	0.552^***^	0.634^***^
	(−6.02)	(−0.33)	(−9.32)	(−3.33)
Controls				
Unemployed	0.686^***^		0.907^***^	
Post-secondary education	1.338^***^		1.374^***^	
Income	1.000^***^		1.000^***^	
Occupation	Y		Y	
Parental controls	Y		Y	
County	Y		Y	
Observations	3,342,797		2,624,073	

The table reports the exponentiated regression coefficients for the competing risks regression with the failure event defined as leaving the parental home to live independently and the competing risk event defined as leaving the parental home to live in a married household. t statistics in parentheses. See appendix Table 7 for full regression output

Overall, the coefficients in Table 2 are at values below 1, which points to reduced subdistribution hazards for the transition to independent living, in both the Middle Eastern and Eastern European immigrant groups and in the second generation, for both males and females. The effect size measure between 0.65 and 0.69 in the ME first generation males, but is lower at 0.51 in generation 2, while the coefficient is higher at 0.82 in generation 2.5. The gap with natives is larger in the ME female population, with coefficients measuring between 0.46 and 0.55 in the first generation, decreasing to 0.38 in generation 2. Comparing these effect sizes to those of the Eastern European sample, they appear to be similar in the first-generation males but diverge in the case of females in generation 1, and both males and females in generation 2.

Looking at the control variables, unemployment decreases the subdistribution hazard, especially for males. Post-secondary education increases the subdistribution hazard and has a similar effect for both males and females, while income has a vanishingly small effect. The addition of individual and family controls only shifts the gap coefficients by a small measure of 0.1 or less, as compared to the unadjusted regression.^3^

**Fig. 1:**
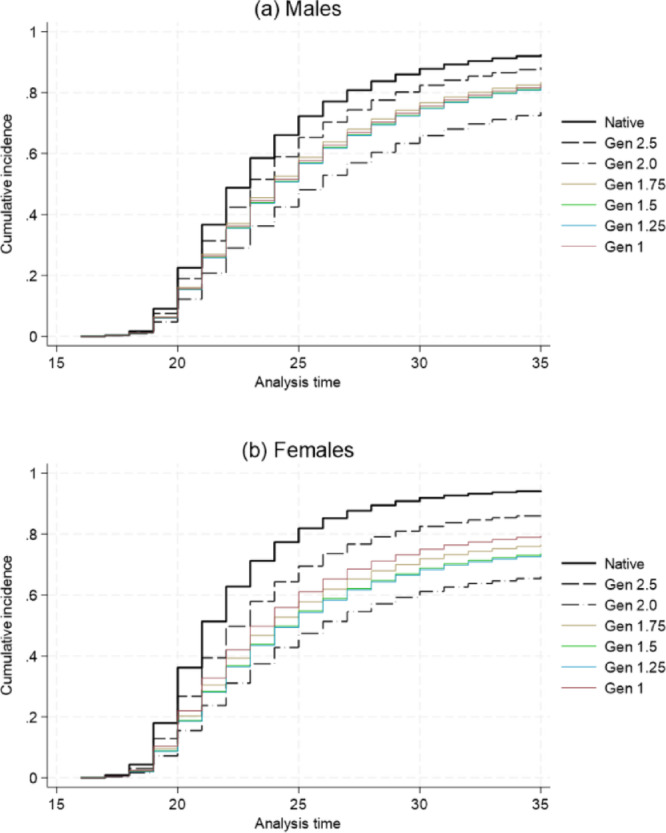
Cumulative incidence functions. The cumulative incidence functions are based on the models controlling for individual controls, parental controls, and county fixed effects

Figure 1 shows the cumulative incidence functions for ME immigrants and the second generation alongside those of the native sample using the models estimated in Table 2, for both males and females. The cumulative incidence functions show the proportion of individuals that have transitioned to living independently by a given age, giving a better view of both the timing of transitions and the proportion eventually making the transition. In the case of native males, over 80% transition by the age of 27. Gen 2.5 matches this group closely, with 80% completing the transition by age 29. The first-generation immigrants transition at a slower rate, reaching 80% at age 31. Meanwhile, the transitions are slowest in generation 2, where only around 60% transition by age 28, and only around 75% transition by the end of the observation period. In the case of females, the gaps are larger, with the share transitioning at less than 80% in the first generation, and only around 65% in generation 2.

Additional results reported in Appendix Table A2 treat the transition to a married household as the main outcome event, with independent living treated as the competing risk. The estimates show that transitions into marriage are more common among the ME-origin groups relative to natives, particularly among females. Across most generational categories, the coefficients for females are higher than those for males, indicating comparatively higher subdistribution hazards of leaving the parental home through marriage. These differences are especially visible in the second generation. Overall, the appendix results complement those in Table 2 by showing that the lower rates of independent living observed among ME-origin youth are accompanied by relatively higher transitions into marriage.

###  The Role of Parental Income

To explore the role of parental income, we estimate the competing risks regression coefficients interacted with parental income quartiles, calculated using the sum of the two parents’ incomes. The results are shown in Table 3. We find that the size of the coefficients varies only modestly across the bottom three income quartiles, while the top income quartile is associated with smaller gaps relative to natives across several generations among males, but less consistently among females. Overall, the gaps with natives remain substantial across all parental income quartiles. This suggests that parental income plays some role in shaping the transition to living independently, particularly at the upper end of the parental income distribution, but does not by itself account for the observed differences.

**Table 3:** Coefficients of competing risks regression according to parental income quartile

	Male				Female			
	(1)				(2)			
Reference	Natives, Quartile 1				Natives, Quartile 1			
Origin	ME				ME			
Quartile	1	2	3	4	1	2	3	4
Generation								
2.5	0.755^**^	1.039	0.842^**^	1.265^***^	0.648^***^	0.766^**^	0.619^***^	0.762^***^
	(−3.03)	(0.47)	(−2.71)	(5.76)	(−4.68)	(−3.09)	(−6.53)	(−6.32)
2	0.483^***^	0.507^***^	0.549^***^	0.819^***^	0.370^***^	0.344^***^	0.367^***^	0.417^***^
	(−23.32)	(−17.95)	(−20.28)	(−8.73)	(−29.87)	(−25.71)	(−28.86)	(−24.73)
1.75	0.725^***^	0.716^***^	0.809^***^	1.025	0.539^***^	0.521^***^	0.470^***^	0.523^***^
	(−7.78)	(−6.69)	(−5.21)	(0.76)	(−12.70)	(−11.36)	(−14.19)	(−14.13)
1.5	0.703^***^	0.746^***^	0.717^***^	0.945	0.512^***^	0.473^***^	0.518^***^	0.403^***^
	(−10.02)	(−6.54)	(−8.18)	(−1.73)	(−16.32)	(−13.69)	(−13.61)	(−10.39)
1.25	0.701^***^	0.737^***^	0.670^***^	0.922	0.484^***^	0.431^***^	0.441^***^	0.452^***^
	(−7.66)	(−4.20)	(−5.78)	(−1.46)	(−12.36)	(−7.85)	(−7.73)	(−8.51)
1	0.764^**^	0.916	0.544^**^	0.853	0.590^***^	0.494^***^	0.320^***^	0.711^**^
	(−2.66)	(−0.58)	(−2.88)	(−1.14)	(−5.38)	(−3.59)	(−4.78)	(−2.98)
Observations	3,315,230				2,602,251			

The table reports the exponentiated regression coefficients according to parental income quartile. The reference group is native Swedes in the bottom income quartile. The regressions control for employment status, post-secondary education, occupation, income, parental controls, and county fixed effects. t statistics in parentheses

### Heterogeneity by Country of Origin

To explore heterogeneity by country of origin, we estimate models that interact country of origin with generation, instead of treating all ME-origin individuals as a single group. This part of the analysis is exploratory and is intended to show whether the broad ME pattern masks important differences across origin groups. Such differences may reflect variation in migration histories, duration of residence, family norms, and the generational composition of the origin groups in Sweden.

**Fig. 2:**
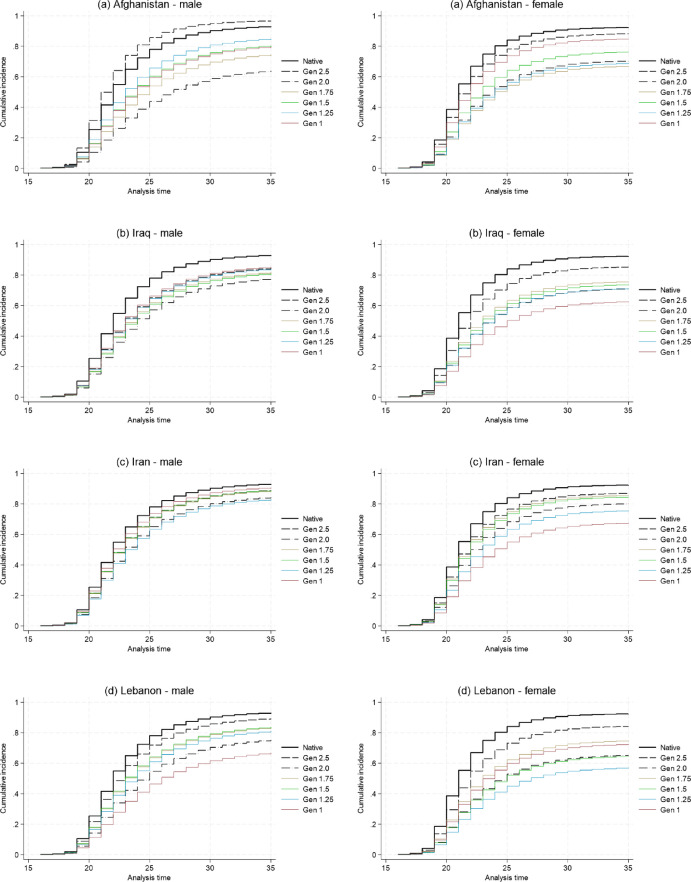

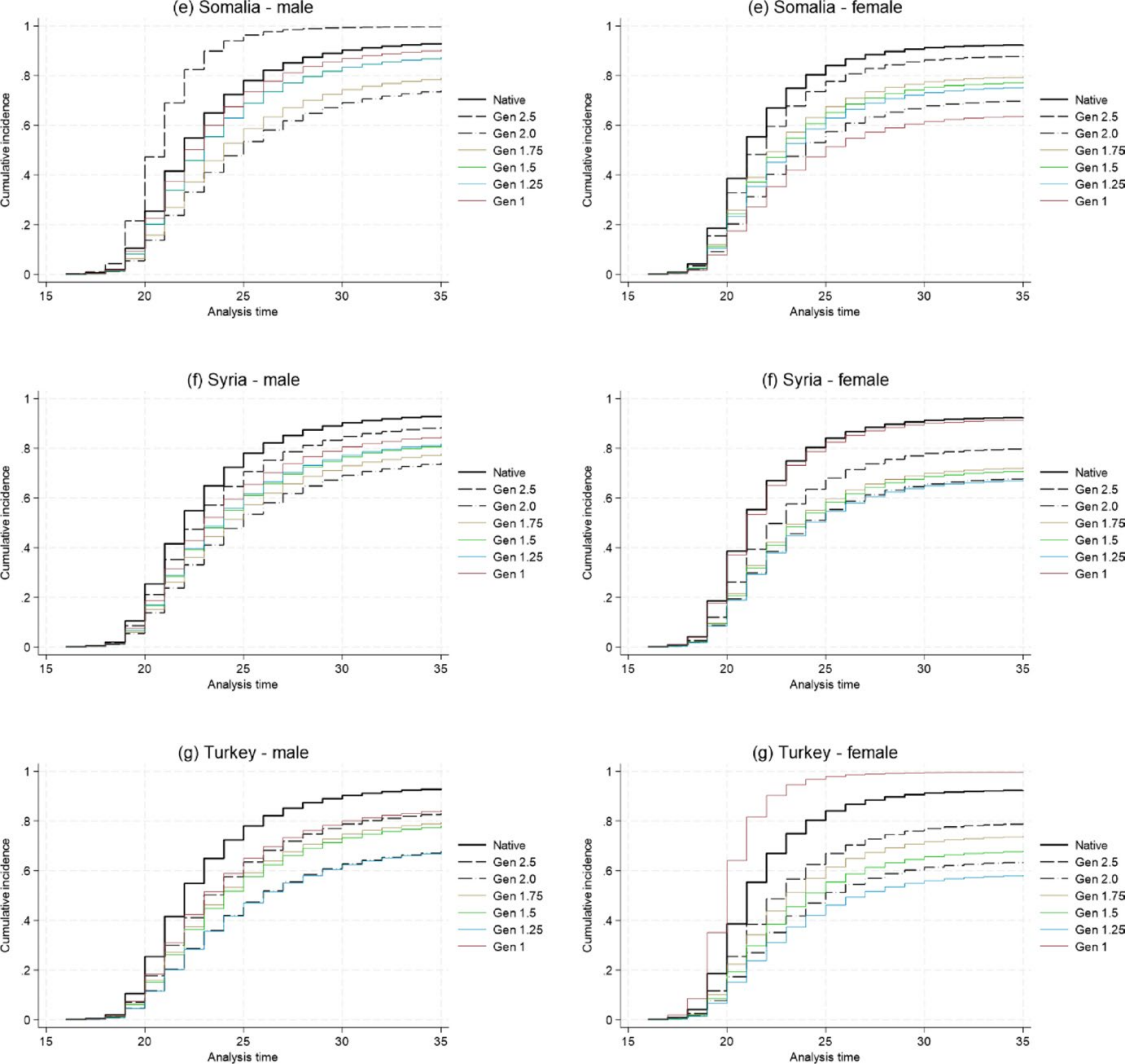
Cumulative incidence functions according to country of origin

We plot the resulting cumulative incidence functions by country of origin for males and females in Fig. 2. We find substantial heterogeneity by country of origin for males, with some countries matching the native trajectories more closely, especially Iran and Iraq. The degree of the divergence in generation 2 differs by country of origin, and appears strongest in the case of Afghanistan, but is present to some extent in most origin countries. Overall, there is less variation by country of origin in the case of females.

### The Role of the Neighbourhood

We test the contribution of two variables to explaining the gaps in transitions between immigrants and natives – the share of natives in the local postcode, and the share of unemployed individuals in the local postcode. Table 4 shows the size of the coefficients of competing risks regressions where we interact with a dummy variable that takes the value 1 if the share of natives is at 75% or more and 0 otherwise. We find significant differences in the size of the coefficients across neighbourhood native shares, in generations 1.5 to 2, for both males and females. In the case of generation 2 males, the effect sizes in neighbourhoods with high native shares is at 0.7 as compared to 0.54 in neighbourhoods with low native shares. The corresponding coefficients are 0.51 and 0.38 in the case of females.

**Table 4:** Coefficients of competing risks regression according to postcode native share

	Male		Female	
	(1)		(2)	
Reference	Natives, ≥ 75%		Natives, ≥ 75%	
Origin	ME	ME	ME	ME
Native share	≥ 75%	< 75%	≥ 75%	< 75%
Generation				
2.5	0.917^*^	1.057	0.777^***^	0.829^**^
	(−2.42)	(0.86)	(−7.10)	(−2.58)
2	0.703^***^	0.544^***^	0.510^***^	0.385^***^
	(−17.17)	(−30.68)	(−29.45)	(−40.98)
1.75	0.894^***^	0.742^***^	0.653^***^	0.524^***^
	(−3.75)	(−9.71)	(−12.08)	(−17.06)
1.5	0.848^***^	0.724^***^	0.609^***^	0.489^***^
	(−5.54)	(−11.91)	(−13.36)	(−14.94)
1.25	0.815^***^	0.759^***^	0.502^***^	0.556^***^
	(−3.88)	(−7.24)	(−9.57)	(−11.04)
1	0.839	0.745^**^	0.670^***^	0.614^***^
	(−1.79)	(−2.74)	(−4.62)	(−4.71)
Observations	3,297,430		2,588,050	

The table reports the exponentiated regression coefficients for the competing risks regressions. The reference group is Sweden natives in postcodes with 75% or more native population. The regressions control for employment status, post-secondary education, occupation, income, parental controls, and county fixed effects. t statistics in parentheses

Table 5 shows the coefficients interacted with a dummy variable that takes the value 1 if the share of unemployment is below 25% and 0 otherwise. The coefficients differ markedly between the two neighbourhood contexts. Males in high unemployment neighbourhoods transition to independent living at substantially lower rates than their counterparts in low unemployment neighbourhoods. The gaps are even larger in the case of females. Overall, the neighbourhood-level unemployment appears to be a much more significant determinant of transition rates as compared to the neighbourhood native share.

**Table 5:** Coefficients from competing risks regression according to postcode unemployment level

	Male		Female	
	(1)		(2)	
Reference	Natives, < 25%		Natives, < 25%	
Origin	ME	ME	ME	ME
Unemployed	< 25%	≥ 25%	< 25%	≥ 25%
Generation				
2.5	0.812^***^	0.409^***^	0.700^***^	0.119^***^
	(−6.07)	(−15.61)	(−9.37)	(−31.19)
2	0.494^***^	0.267^***^	0.341^***^	0.078^***^
	(−45.33)	(−36.90)	(−52.47)	(−50.33)
1.75	0.667^***^	0.354^***^	0.455^***^	0.102^***^
	(−16.99)	(−24.80)	(−24.39)	(−40.20)
1.5	0.626^***^	0.346^***^	0.403^***^	0.096^***^
	(−20.95)	(−26.41)	(−19.23)	(−42.37)
1.25	0.631^***^	0.332^***^	0.383^***^	0.090^***^
	(−12.89)	(−20.51)	(−15.50)	(−34.62)
1	0.624^***^	0.345^***^	0.584^***^	0.092^***^
	(−4.82)	(−10.55)	(−5.66)	(−22.94)
Observations	3,297,430		2,588,050	

The table reports the exponentiated regression coefficients for the competing risks regressions. The reference group is Sweden natives in postcodes with less than 25% unemployment level. The regressions control for employment status, post-secondary education, occupation, income, parental controls, and county fixed effects. t statistics in parentheses

We interact the two neighbourhood variables in Table 6, further splitting the sample according to both neighbourhood native share and neighbourhood unemployment level. We find the largest gaps in high-unemployment neighbourhoods, in both low- and high- native share neighbourhoods. In the case of females, effect sizes are particularly stark in these neighbourhoods, measuring at around 0.1.

**Table 6:** Coefficients from competing risks regression according to unemployment level and native share at the zip code level

	Male				Female			
	(1)				(2)			
Reference	Natives, Unemployed < 25%, Natives ≥ 75%				Natives, Unemployed < 25%, Natives ≥ 75%			
Origin	ME	ME	ME	ME	ME	ME	ME	ME
Unemployed	< 25%	≥ 25%	< 25%	≥ 25%	< 25%	≥ 25%	< 25%	≥ 25%
Native share	≥ 75%	≥ 75%	< 75%	< 75%	≥ 75%	≥ 75%	< 75%	< 75%
Generation								
2.5	0.916^*^	0.440^***^	0.937	0.611^***^	0.795^***^	0.132^***^	0.762^**^	0.168^***^
	(−2.06)	(−12.21)	(−0.83)	(−4.55)	(−5.07)	(−26.82)	(−3.10)	(−14.91)
2	0.674^***^	0.363^***^	0.513^***^	0.294^***^	0.469^***^	0.103^***^	0.336^***^	0.0865^***^
	(−16.22)	(−22.86)	(−27.78)	(−27.77)	(−25.82)	(−40.29)	(−37.17)	(−42.84)
1.75	0.851^***^	0.463^***^	0.704^***^	0.386^***^	0.584^***^	0.135^***^	0.464^***^	0.110^***^
	(−4.33)	(−14.28)	(−9.15)	(−16.91)	(−11.49)	(−30.12)	(−15.51)	(−31.18)
1.5	0.788^***^	0.455^***^	0.681^***^	0.384^***^	0.522^***^	0.130^***^	0.418^***^	0.105^***^
	(−6.36)	(−14.96)	(−11.58)	(−18.54)	(−12.68)	(−30.80)	(−11.88)	(−35.10)
1.25	0.732^***^	0.458^***^	0.740^***^	0.375^***^	0.403^***^	0.107^***^	0.466^***^	0.112^***^
	(−4.67)	(−9.52)	(−6.33)	(−14.58)	(−8.26)	(−22.21)	(−9.42)	(−27.10)
1	0.838	0.403^***^	0.580^**^	0.431^***^	0.738^*^	0.113^***^	0.591^**^	0.111^***^
	(−1.31)	(−6.19)	(−3.18)	(−6.10)	(−2.25)	(−15.31)	(−3.24)	(−15.73)
Observations	3,297,430				2,588,050			

The table reports the exponentiated regression coefficients for the competing risks regressions. The reference group is Sweden natives in postcodes with 75% or more native population and unemployment level below 25%. The regressions control for employment status, post-secondary education, occupation, income, parental controls, and county fixed effects. t statistics in parentheses

## Discussion and Conclusion

Our results underscore three central findings: first, youth of ME background are less likely to leave the parental home for independent living than their native Swedish peers; second, these differences are especially pronounced among women and among the second generation; and third, neighbourhood context—particularly local unemployment—is strongly associated with lower rates of transition to independent living, whereas parental economic resources play a more limited but still visible role, especially at the upper end of the parental income distribution.

The finding that ME-origin youth are more likely to transition directly into marriage than into independent living is consistent with evidence from origin countries, where home-leaving is closely tied to marital status, particularly for women (Koç, 2007; Sonneveld, 2019). At the same time, our results show that the majority of ME-origin youth in Sweden still leave for independent living, which contrasts with the dominant pattern in their countries of origin. This suggests partial adaptation to destination-country norms alongside persistent differences in pathways. Prior studies similarly point to this mixed pattern. In the Netherlands, Turkish and Moroccan youth tend to leave home earlier than natives, yet their departures are more often linked to marriage or union formation rather than to independent living (Zorlu & Mulder, 2011). In Canada, generational and visible minority differences further highlight this complexity, as second-generation youth may converge with natives in timing while pathways out of the parental home continue to diverge (Haan et al., 2023). Our findings fit squarely within this broader evidence of simultaneous adaptation and divergence. In the Swedish case, we show that second-generation ME youth, particularly women, are less likely to leave independently than their first-generation peers, while the 2.5 generation more closely resembles natives.

These patterns speak to the idea that assimilation does not necessarily follow a simple, linear trajectory. The so-called “second-generation paradox” highlights that outcomes may stagnate or even diverge across generations in certain domains. In Sweden, recent studies document similar dynamics, including higher unemployment risks among the second generation (Aradhya et al., 2023) and a reversal of the mortality advantage often observed among first-generation immigrants (Wallace, 2022). Our findings add to this literature by showing that transitions out of the parental home do not follow a monotonic generational gradient. Rather than moving progressively closer to native Swedish patterns, second-generation youth with two foreign-born parents are, in several cases, less likely to leave the parental home in ways that mirror the native population than some first-generation youth who arrived in Sweden during childhood. At the same time, while this pattern is consistent with a second-generation paradox in the domain of home-leaving, it should not be interpreted as direct evidence of lower integration, as part of the observed differences may reflect unmeasured heterogeneity in parental migration backgrounds across generations.

The mechanisms underlying these patterns are likely multifaceted and cannot be identified directly in our data. Our results are consistent with the idea that family norms around co-residence and marriage persist across generations (de Valk & Liefbroer, 2007), while also highlighting the importance of neighbourhood context. Immigrant-dense areas may provide important support networks while sustaining expectations around remaining in the parental home (McAvay & Pailhé, 2022). However, our findings suggest that structural constraints—particularly local labour market conditions—play an even more prominent role. The strong and consistent association between postcode-level unemployment and delayed transitions to independent living indicates that economic opportunities shape the feasibility of residential independence, interacting with, rather than replacing, family and community influences.

Gender differences further reinforce this interpretation. Women of ME origin display larger gaps relative to native Swedes than men, especially in the second generation. This aligns with earlier research showing that young women in migrant families often face stricter expectations around family responsibility, modesty, and protection (Berry et al., 2006; Gebel & Heyne, 2014). Remaining in the parental home until marriage may therefore reflect not only preferences but also constraints shaped by family expectations and community norms. For men, by contrast, expectations may place greater emphasis on achieving financial stability prior to household formation, pointing to different but equally structured pathways into adulthood.

The contrast between the second generation and the 2.5 generation further highlights the importance of household-level socialization. Youth with one Swedish-born parent display home-leaving patterns much closer to those of natives, consistent with research linking intermarriage to faster assimilation (Tegunimataka, 2021). This suggests that exposure to mixed cultural environments within the household may weaken the persistence of origin-country norms and facilitate earlier transitions to independent living. By contrast, in households where both parents are foreign-born, these norms may be more strongly reinforced.

Taken together, these findings contribute to the literature by showing that pathways out of the parental home remain stratified by origin, gender, and neighbourhood context, even within a welfare-state setting characterized by relatively early home-leaving. Rather than reflecting a simple process of convergence, transitions to residential independence exhibit a segmented pattern shaped by the interaction of cultural norms and structural constraints. At the same time, the limited explanatory power of parental economic resources suggests that these differences cannot be reduced to socioeconomic disadvantage alone.

From a policy perspective, our findings highlight the importance of structural conditions—particularly labour market opportunities and housing access—in shaping youth transitions. While Sweden’s welfare institutions support early independence in general, local inequalities in employment and residential environments may sustain differences in life-course trajectories. Policies aimed at improving access to affordable housing, reducing spatial concentrations of disadvantage, and supporting young adults’ labour market integration may therefore be central to enabling more equal transitions to independent living.

Finally, several limitations point to directions for future research. Our competing risks framework captures only the first transition out of the parental home and does not account for return moves or more complex residential trajectories. In addition, the data do not allow us to directly observe cohabitation or fully account for heterogeneity in parental migration histories. Future research could extend this work by examining repeated transitions (“boomerang” behaviour; Olofsson et al., 2020), incorporating richer measures of partnership formation, and using qualitative and survey-based approaches to better understand how young adults navigate the interplay between family expectations, community norms, and structural constraints.

## Figures and Tables

**Fig. 3:**
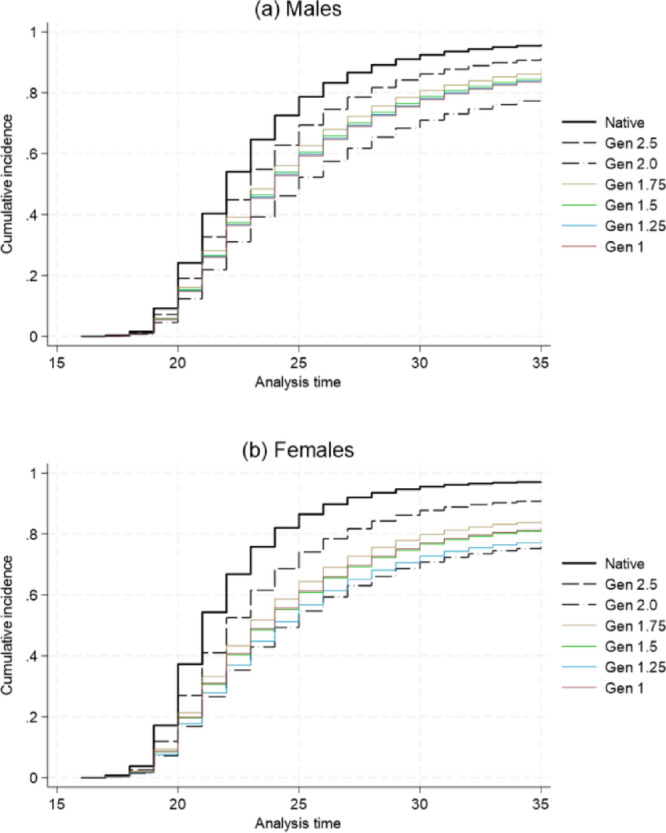
Cumulative incidence functions (without parental controls). The cumulative incidence functions are based on the models controlling without parental controls

**Fig. 4:**
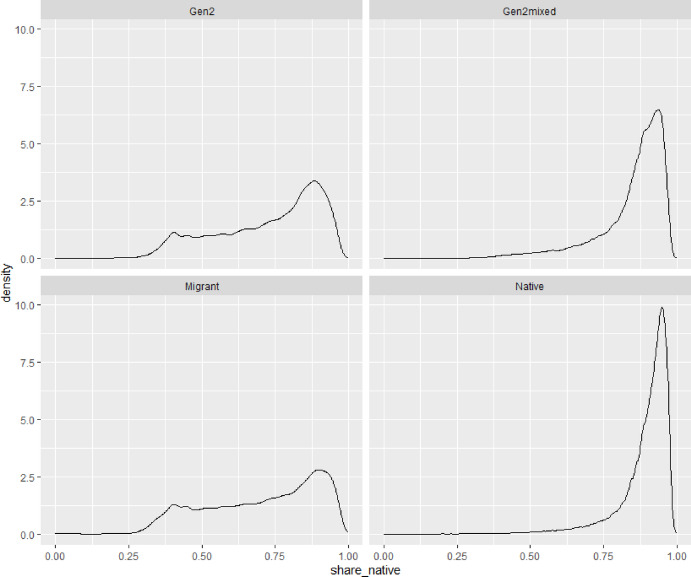
Distribution of individuals in the sample according to the share of native Swedes in the same ZIP code. The figures plot the distribution of individuals according to the share of native individuals in the local postcode, according to migrant status

## Data Availability

Data was made available by Statistics Sweden.

## Appendix

See Figs. 3, 4 and Tables 7, 8, 9 and 10.

**Table 7:** Full regression output with the transition into independent living as the main failure event

	(1)	(2)
	Males	Females
Native Swedes	1	1
	(.)	(.)
ME_2.5	0.824^***^	0.695^***^
	(−6.81)	(−12.16)
ME_2.0	0.511^***^	0.376^***^
	(−52.88)	(−62.45)
ME_1.75	0.690^***^	0.505^***^
	(−19.79)	(−28.20)
ME_1.5	0.657^***^	0.464^***^
	(−23.45)	(−24.81)
ME_1.25	0.653^***^	0.458^***^
	(−15.10)	(−19.30)
ME_1.0	0.670^***^	0.552^***^
	(−6.02)	(−9.32)
EEURO_2.5	0.846^***^	0.836^***^
	(−8.58)	(−9.28)
EEURO_2.0	0.701^***^	0.629^***^
	(−15.62)	(−18.65)
EEURO_1.75	0.770^***^	0.696^***^
	(−6.84)	(−8.54)
EEURO_1.5	0.648^***^	0.634^***^
	(−10.02)	(−9.22)
EEURO_1.25	0.606^***^	0.576^***^
	(−8.85)	(−9.05)
EEURO_1.0	0.934	0.634^***^
	(−0.33)	(−3.33)
Post secondary education	1.338^***^	1.374^***^
	(71.81)	(72.19)
Unemployed	0.686^***^	0.907^***^
	(−52.00)	(−5.09)
Unemployed mother	1.026^***^	1.066^***^
	(5.10)	(11.03)
Unemployed father	0.996	1.010
	(−0.86)	(1.93)
Occupation		
Farming, industrial, and transport workers	1	1
	(.)	(.)
Managers and officials	1.054	0.995
	(1.60)	(−0.11)
Military Personnel	1.030	1.127^*^
	(1.60)	(2.22)
Professionals	1.029^***^	1.008
	(4.28)	(0.81)
Service workers	0.948^***^	1.034^***^
	(−11.67)	(3.58)
Unknown	0.878^***^	0.995
	(−28.27)	(−0.49)
Mother’s occupation		
Farming, industrial, and transport workers	1	1
	(.)	(.)
Managers and officials	1.012	0.917^***^
	(1.17)	(−8.35)
Professionals	1.005	0.895^***^
	(0.60)	(−14.10)
Service workers	0.995	0.952^***^
	(−0.65)	(−6.76)
Unknown	1.054^***^	0.915^***^
	(3.80)	(−6.02)
Father’s occupation		
Farming, industrial, and transport workers	1	1
	(.)	(.)
Managers and officials	1.029^***^	0.945^***^
	(5.50)	(−10.77)
Professionals	1.027^***^	0.927^***^
	(6.71)	(−19.64)
Service workers	1.016^***^	0.966^***^
	(3.30)	(−7.13)
Unknown	0.975^*^	0.878^***^
	(−2.11)	(−10.62)
Income	1.000^***^	1.000^***^
	(5.98)	(11.98)
Mother’s income	1.000^***^	1.000^***^
	(5.02)	(−7.02)
Father’s income	1.000^***^	1.000^***^
	(6.93)	(−7.97)
County		
Stockholm	1	1
	(.)	(.)
Uppsala	1.382^***^	1.456^***^
	(39.01)	(44.56)
Södermanland	1.239^***^	1.206^***^
	(20.57)	(16.83)
Östergötland	1.635^***^	1.539^***^
	(63.83)	(54.26)
Jönköping	1.136^***^	1.129^***^
	(14.74)	(13.25)
Kronoberg	1.317^***^	1.347^***^
	(25.29)	(25.97)
Kalmar	1.266^***^	1.281^***^
	(23.59)	(23.36)
Gotland	0.907^***^	0.849^***^
	(−5.08)	(−8.04)
Blekinge	1.318^***^	1.181^***^
	(22.27)	(12.25)
Skåne	1.179^***^	1.197^***^
	(30.84)	(32.69)
Halland	0.969^***^	0.944^***^
	(−3.48)	(−5.89)
Västra Götaland	1.146^***^	1.164^***^
	(28.00)	(29.76)
Värmland	1.318^***^	1.340^***^
	(29.52)	(30.14)
Örebro	1.409^***^	1.431^***^
	(37.03)	(37.79)
Västmanland	1.308^***^	1.269^***^
	(25.80)	(21.52)
Dalarna	1.105^***^	1.087^***^
	(10.40)	(8.09)
Gävleborg	1.229^***^	1.224^***^
	(21.32)	(19.82)
Västernorrland	1.151^***^	1.220^***^
	(14.04)	(18.50)
Jämtland	1.087^***^	1.192^***^
	(6.25)	(12.81)
Västerbotten	1.519^***^	1.612^***^
	(47.68)	(54.28)
Norrbotten	1.301^***^	1.275^***^
	(26.98)	(22.27)
Observations	3,342,797	2,624,073

Exponentiated coefficients; *t* statistics in parentheses

^*^ *p* < 0.05, ^**^ *p* < 0.01, ^***^ *p* < 0.001

**Table 8:** Full regression output with the transition into marriage as the main failure event

	(1)	(2)
	Males	Females
Native Swedes	1	1
	(.)	(.)
ME_2.5	2.864^***^	4.202^***^
	(4.78)	(9.42)
ME_2.0	9.647^***^	11.97^***^
	(43.06)	(56.00)
ME_1.75	7.008^***^	9.831^***^
	(20.97)	(32.04)
ME_1.5	8.825^***^	11.33^***^
	(28.02)	(35.58)
ME_1.25	9.812^***^	16.54^***^
	(21.98)	(30.36)
ME_1.0	9.725^***^	8.477^***^
	(9.22)	(9.76)
EEURO_2.5	1.299	0.928
	(1.11)	(−0.31)
EEURO_2.0	2.067^***^	2.466^***^
	(3.64)	(5.72)
EEURO_1.75	5.068^***^	5.132^***^
	(7.06)	(8.10)
EEURO_1.5	8.381^***^	6.438^***^
	(10.66)	(9.57)
EEURO_1.25	3.767^***^	7.291^***^
	(3.95)	(8.33)
EEURO_1.0	5.224	2.264
	(1.69)	(0.81)
Post secondary education	1.752^***^	1.906^***^
	(13.69)	(18.45)
Unemployed	0.462^***^	1.031
	(−16.82)	(0.71)
Unemployed mother	1.348^***^	1.147^**^
	(5.87)	(2.97)
Unemployed father	0.997	1.010
	(−0.06)	(0.22)
Occupation		
Farming, industrial, and transport workers	1	1
	(.)	(.)
Managers and officials	1.937^*^	2.589^**^
	(2.34)	(2.68)
Military Personnel	0.213^**^	3.67e−08^***^
	(−2.67)	(−131.79)
Professionals	1.571^***^	2.616^***^
	(6.18)	(8.41)
Service workers	0.903	1.284^*^
	(−1.85)	(2.36)
Unknown	0.644^***^	1.173
	(−7.84)	(1.49)
Mother’s occupation		
Farming, industrial, and transport workers	1	1
	(.)	(.)
Managers and officials	0.869	1.085
	(−0.93)	(0.66)
Professionals	1.049	0.996
	(0.47)	(−0.05)
Service workers	1.091	1.193^*^
	(0.87)	(2.08)
Unknown	2.044^***^	2.149^***^
	(6.50)	(7.70)
Father’s occupation		
Farming, industrial, and transport workers	1	1
	(.)	(.)
Managers and officials	0.867	0.879^*^
	(−1.78)	(−1.97)
Professionals	0.932	0.922
	(−1.28)	(−1.81)
Service workers	1.169^**^	1.176^***^
	(2.65)	(3.49)
Unknown	1.899^***^	1.378^***^
	(9.68)	(5.06)
Income	1.000^***^	1.000^***^
	(8.12)	(12.88)
Mother’s income	1.000	1.000
	(−0.65)	(−1.67)
Father’s income	1.000	1.000
	(−0.51)	(−0.76)
County		
Stockholm	1	1
	(.)	(.)
Uppsala	0.825^*^	0.728^***^
	(−2.14)	(−3.98)
Södermanland	0.856	0.924
	(−1.35)	(−0.82)
Östergötland	0.832^*^	0.752^***^
	(−2.14)	(−3.60)
Jönköping	1.692^***^	1.902^***^
	(7.25)	(10.75)
Kronoberg	0.857	0.645^***^
	(−1.19)	(−3.36)
Kalmar	0.931	0.743^*^
	(−0.60)	(−2.54)
Gotland	0.512^*^	0.458^**^
	(−2.20)	(−2.79)
Blekinge	0.638^*^	0.545^***^
	(−2.52)	(−3.38)
Skåne	0.871^*^	0.858^**^
	(−2.34)	(−3.02)
Halland	0.721^**^	0.812^*^
	(−2.77)	(−2.08)
Västra Götaland	1.016	1.044
	(0.32)	(1.03)
Värmland	0.629^***^	0.581^***^
	(−3.54)	(−4.55)
Örebro	0.969	0.899
	(−0.32)	(−1.27)
Västmanland	0.867	0.792^*^
	(−1.30)	(−2.30)
Dalarna	0.647^***^	0.679^**^
	(−3.30)	(−3.26)
Gävleborg	0.740^*^	0.820
	(−2.44)	(−1.87)
Västernorrland	0.887	0.732^*^
	(−0.96)	(−2.55)
Jämtland	0.652^*^	0.453^***^
	(−2.30)	(−4.09)
Västerbotten	0.802^*^	0.606^***^
	(−2.01)	(−4.67)
Norrbotten	0.999	1.050
	(−0.01)	(0.48)
Observations	3,342,797	2,624,073

Exponentiated coefficients; *t* statistics in parentheses

^*^ *p* < 0.05, ^**^ *p* < 0.01, ^***^ *p* < 0.001

**Table 9:** Coefficients of competing risks regression without control variables

	Male		Female	
	(1)		(2)	
Reference	Natives		Natives	
Origin	ME	EEURO	ME	EEURO
Generation				
2.5	0.766^***^	0.800^***^	0.674^***^	0.797^***^
	(−9.30)	(−11.47)	(−13.76)	(−11.96)
2	0.479^***^	0.644^***^	0.395^***^	0.606^***^
	(−62.01)	(−19.29)	(−67.29)	(−21.49)
1.75	0.637^***^	0.703^***^	0.514^***^	0.665^***^
	(−24.83)	(−9.25)	(−29.06)	(−9.62)
1.5	0.601^***^	0.600^***^	0.468^***^	0.589^***^
	(−29.38)	(−11.98)	(−33.99)	(−10.79)
1.25	0.589^***^	0.520^***^	0.418^***^	0.502^***^
	(−19.44)	(−11.67)	(−22.19)	(−11.15)
1	0.582^***^	0.833	0.474^***^	0.562^***^
	(−8.75)	(−0.87)	(−11.59)	(−4.87)
Controls				
Unemployment	N		N	
Post−secondary education	N		N	
Occupation	N		N	
Income	N		N	
Parental controls	N		N	
County FE’s	N		N	
Observations	3,342,797		2,624,073	

The table reports the regression coefficients for the competing risks regression with the failure event defined as leaving the parental home to live independently and the competing risk event defined as leaving the parental home to live in a married household

**Table 10:** Coefficients of competing risks regression with municipality fixed effects

	Male		Female	
	(1)		(2)	
Reference	Natives		Natives	
Origin	ME	EEURO	ME	EEURO
Generation				
2.5	0.732^***^	0.809^***^	0.623^***^	0.797^***^
	(−10.35)	(−10.13)	(−15.01)	(−10.98)
2	0.443^***^	0.631^***^	0.333^***^	0.556^***^
	(−60.05)	(−19.19)	(−67.65)	(−22.38)
1.75	0.575^***^	0.679^***^	0.435^***^	0.591^***^
	(−28.11)	(−9.64)	(−34.15)	(−11.56)
1.5	0.552^***^	0.586^***^	0.397^***^	0.567^***^
	(−31.59)	(−11.68)	(−33.59)	(−11.15)
1.25	0.569^***^	0.565^***^	0.405^***^	0.580^***^
	(−19.37)	(−9.58)	(−21.84)	(−8.41)
1	0.652^***^	0.513^**^	0.514^***^	0.557^**^
	(−5.47)	(−2.76)	(−8.71)	(−2.84)
Controls				
Unemployment	Y		Y	
Post−secondary education	Y		Y	
Occupation	Y		Y	
Income	Y		Y	
Parental controls	Y		Y	
Municipality FE’s	Y		Y	
Observations	3,385,303		2,657,346	

The table reports the regression coefficients for the competing risks regression with the failure event defined as leaving the parental home to live independently and the competing risk event defined as leaving the parental home to live in a married household

## Footnotes

1 We retain the first-generation group arriving at age 17 (G1) in the analysis in order to preserve comparability across generational categories and maximise sample size. However, we acknowledge that this group is likely to be more selected than younger-arriving cohorts, particularly because inclusion requires non-missing information on parental characteristics. This may induce selection, as individuals arriving at older ages with linked parental information are a non-random subset of all immigrants. In practice, G1 constitutes a relatively small share of the sample, and results are not driven by its inclusion.

2 The 75% cutoff for the postcode-level immigrant share is chosen to reflect a meaningful division in the distribution of neighbourhood composition. Specifically, examining the distribution of immigrants and second-generation individuals across neighbourhood native shares (Appendix Figure 4) shows that 75% lies close to the midpoint of these distributions. This implies that the threshold separates individuals living in relatively native-dominated neighbourhoods from those in more mixed or immigrant-concentrated settings in a way that is empirically grounded rather than arbitrary. Using this cutoff therefore provides a transparent and data-driven way to distinguish between “high native share” and “low native share” environments, while ensuring sufficient sample sizes on both sides of the threshold for statistical analysis. The 25% cutoff point for the share unemployed was chosen on a similar basis.

3 See appendix Table 9 for unadjusted estimates. The results are robust to using municipality fixed effects in Table 10.
